# Traumatic intraventricular haemorrhages: clinical indicators determining morbidity and mortality in paediatric patients

**DOI:** 10.1007/s00431-026-07054-7

**Published:** 2026-05-21

**Authors:** Zeynep Ölmez Mart, Emel Ulusoy, Bahar Uyuşkan, İlknur Akansu, Anıl Er, Koray Ur, Öznur Eser, Merve Eraslan Canbeldek, Özge Günal, Murat Duman

**Affiliations:** 1 Faculty of Medicine, Department of Pediatrics, Division of Pediatric Emergency Care, Dokuz Eylul University, Izmir, Turkey; 2 Faculty of Medicine, Department of Pediatrics, Dokuz Eylul University, Izmir, Turkey; 3 Department of Pediatrics, Buca Seyfi Demirsoy Training and Research Hospital, Izmir, Turkey; 4 Faculty of Medicine, Department of Neurosurgery, Dokuz Eylul University, Izmir, Turkey

**Keywords:** Trauma, Traumatic brain injury, Intraventricular haemorrhage, Children

## Abstract

Intracranial haemorrhages and traumatic brain injury are significant causes of morbidity and mortality in paediatric patients. Traumatic intraventricular haemorrhage (tIVH) is the least common type of intracranial haemorrhage and typically occurs in association with other intracranial injuries. This study aimed to describe the clinical characteristics of children diagnosed with tIVH, and to identify risk factors associated with poor prognosis. Paediatric patients diagnosed with tIVH in the paediatric emergency department between January 2010 and December 2024 were retrospectively reviewed. Demographic and clinical characteristics, laboratory and imaging results, treatment modalities, and outcomes were analysed. Neurological status at discharge was assessed using the Glasgow Outcome Scale–Extended (GOSE). Among the 785 patients with traumatic intracranial haemorrhage, tIVH was identified in 41 (5.2%). Most patients were male (68.3%), with a median age of 12.8 years (6.6–15.7). The most common mechanism of injury was traffic-related trauma (78.1%). Altered consciousness was observed in 87.8% (*n* = 36), convulsive seizures in 17.1% (*n* = 7), and a Glasgow Coma Scale (GCS) score below 9 on admission in 80.5% (*n* = 33). Isolated tIVH was observed in only three patients (7.3%). Overall, 82.9% of patients (*n* = 34) required admission to the intensive care unit, and the mortality rate was 34.1% (*n* = 14). Only 31.6% of patients achieved a good neurological outcome; notably, all patients with isolated tIVH had favourable outcomes. Low admission GCS, coagulopathy, concomitant subdural haemorrhage, and cerebral herniation were significantly associated with both poor neurological outcome and mortality. In addition, bilateral haemorrhage, cerebral oedema, and midline shift were associated with poor neurological outcome, whereas fourth ventricular haemorrhage, concomitant skull fracture, maxillofacial trauma, and abdominal trauma were significantly associated with mortality (*p* < 0.05).

*Conclusion*: Low admission GCS, coagulopathy, and associated cranial or extracranial injuries are strong predictors of poor prognosis in pediatric tIVH, whereas isolated tIVH is associated with favourable neurological outcomes. Early identification of high-risk features may improve outcomes in pediatric tIVH.

**Table:** 

**What is Known:** *• Traumatic intraventricular haemorrhage (tIVH) is a rare complication of paediatric traumatic brain injury, typically resulting from high-energy trauma such as traffic accidents, and frequently coexists with other severe intracranial injuries.* *• While isolated tIVH is known to follow a favourable and self-limited course, the presence of tIVH in the context of multitrauma is generally associated with high rates of morbidity and mortality.* **What is New:** *• This study demonstrates that poor neurological outcomes and mortality in paediatric tIVH are driven primarily by the severity of concomitant injuries rather than the tIVH itself.* *• Specific clinical and radiological features, such as coagulopathy, concomitant subdural haemorrhage, bilateral haemorrhagic involvement, midline shift, cerebral oedema, fourth ventricular involvement, and concurrent abdominal trauma, were identified as strong prognostic markers in this population.*

**What is Known:**

*• Traumatic intraventricular haemorrhage (tIVH) is a rare complication of paediatric traumatic brain injury, typically resulting from high-energy trauma such as traffic accidents, and frequently coexists with other severe intracranial injuries.*

*• While isolated tIVH is known to follow a favourable and self-limited course, the presence of tIVH in the context of multitrauma is generally associated with high rates of morbidity and mortality.*

**What is New:**

*• This study demonstrates that poor neurological outcomes and mortality in paediatric tIVH are driven primarily by the severity of concomitant injuries rather than the tIVH itself.*

*• Specific clinical and radiological features, such as coagulopathy, concomitant subdural haemorrhage, bilateral haemorrhagic involvement, midline shift, cerebral oedema, fourth ventricular involvement, and concurrent abdominal trauma, were identified as strong prognostic markers in this population.*

## Introduction

Traumatic brain injury (TBI) and intracranial haemorrhages are among the most common causes of morbidity and mortality in paediatric patients [1]. Intracranial haemorrhages are classified according to their anatomical location as epidural, subdural, subarachnoid, intraparenchymal, and intraventricular haemorrhages [2]. Intraventricular haemorrhage is the rarest form of intracranial haemorrhage following trauma and is often associated with severe head trauma and concomitant intracranial injuries, which complicates the interpretation of its independent prognostic significance. Accordingly, it remains unclear whether tIVH independently contributes to poor outcomes or primarily reflects the severity of associated injuries.

In this study, we aimed to describe the epidemiological and clinical characteristics of paediatric patients diagnosed with traumatic intraventricular haemorrhage (tIVH) and to characterise clinical features of paediatric tIVH and identify indicators associated with poor outcomes in real-world settings where tIVH is rarely isolated.

## Materials and methods

Paediatric patients aged 0–18 years who presented to the Paediatric Emergency Department of Dokuz Eylul University Faculty of Medicine with head trauma and were diagnosed with tIVH between January 2010 and December 2024 were retrospectively analysed. Patients with underlying haematological disorders predisposing to bleeding and those for whom diagnostic imaging at presentation was unavailable were excluded.

Demographic data, clinical characteristics at presentation, laboratory and imaging findings, medical and surgical treatments, and outcomes were recorded. The short-time neurological outcome was evaluated based on survival and neurological status at the time of discharge. Neurological outcomes were assessed using the Glasgow Outcome Scale–Extended (GOSE), with scores of 1–5 classified as poor neurological outcome and scores of 6–8 as good neurological outcome [3, 4].

Coagulation parameters at presentation were evaluated, and acute traumatic coagulopathy (ATC) was defined as an age-adjusted prolongation of the prothrombin time and activated partial thromboplastin time, or an international normalized ratio (INR) greater than 1.2 [5]. Patients were additionally assessed using the International Society on Thrombosis and Haemostasis (ISTH) disseminated intravascular coagulation (DIC) scoring system, with scores of 5 or greater indicating DIC [6].

The study protocol was conducted in accordance with the principles outlined in the Declaration of Helsinki. Ethical approval was obtained from the Clinical Research Ethics Committee of Dokuz Eylul University. The study was supported by the Dokuz Eylul University Scientific Research Projects Unit (2025/28—09).

Statistical analyses were performed using SPSS (Statistical Package for the Social Sciences) version 23.0 for Windows. Data distribution was assessed using the Shapiro–Wilk test. Normally distributed variables were expressed as mean ± standard deviation, while non-normally distributed variables were presented as median and interquartile range (25th–75th percentiles). Student’s *t*-test was used for comparisons of normally distributed continuous variables, and the Mann–Whitney *U* test for non-normally distributed variables. Categorical variables were analysed using the chi-square test or Fisher’s exact test, as appropriate. A *p-value* < 0.05 was considered statistically significant.

In a post hoc power analysis based on the multicentre study published by the PECARN study group in 2012, the statistical power of the present study was calculated to be 84.88% with a 95% confidence interval [7].

## Results

**Fig. 1:**
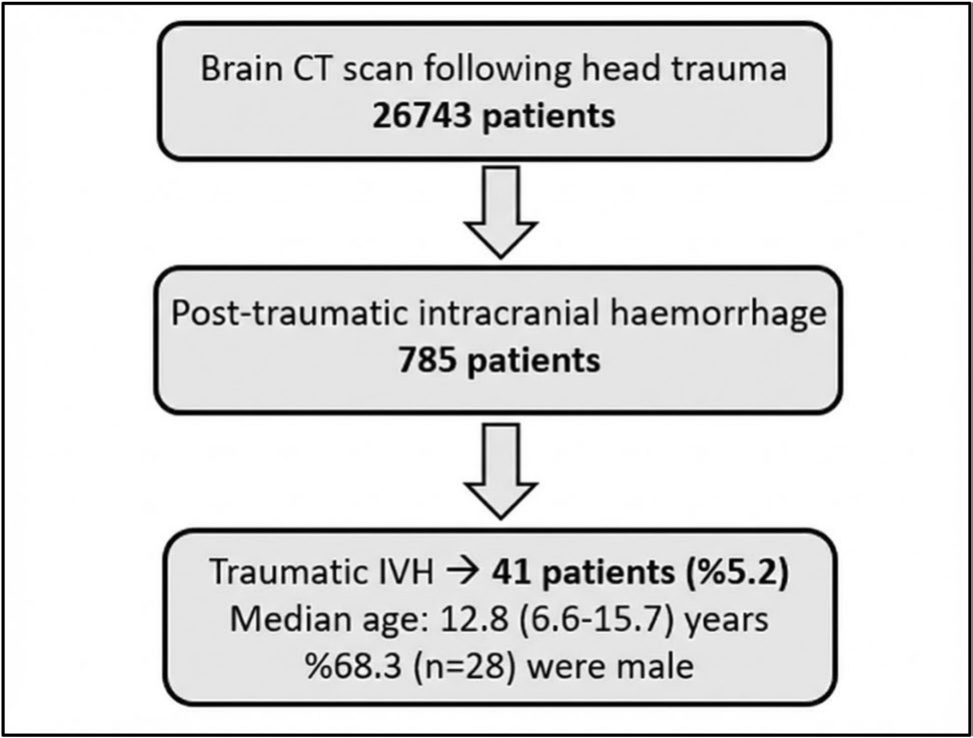
Diagram of patient enrolment

A total of 26,743 paediatric patients presenting to the Paediatric Emergency Department with head trauma underwent cranial computed tomography (CT). Intracranial haemorrhage was detected in 785 patients, of whom 41 (5.2%) had tIVH. The median age of patients with tIVH was 12.8 years (6.6–15.7), and 68.3% were male (n = 28) (Fig. 1).

**Fig. 2:**
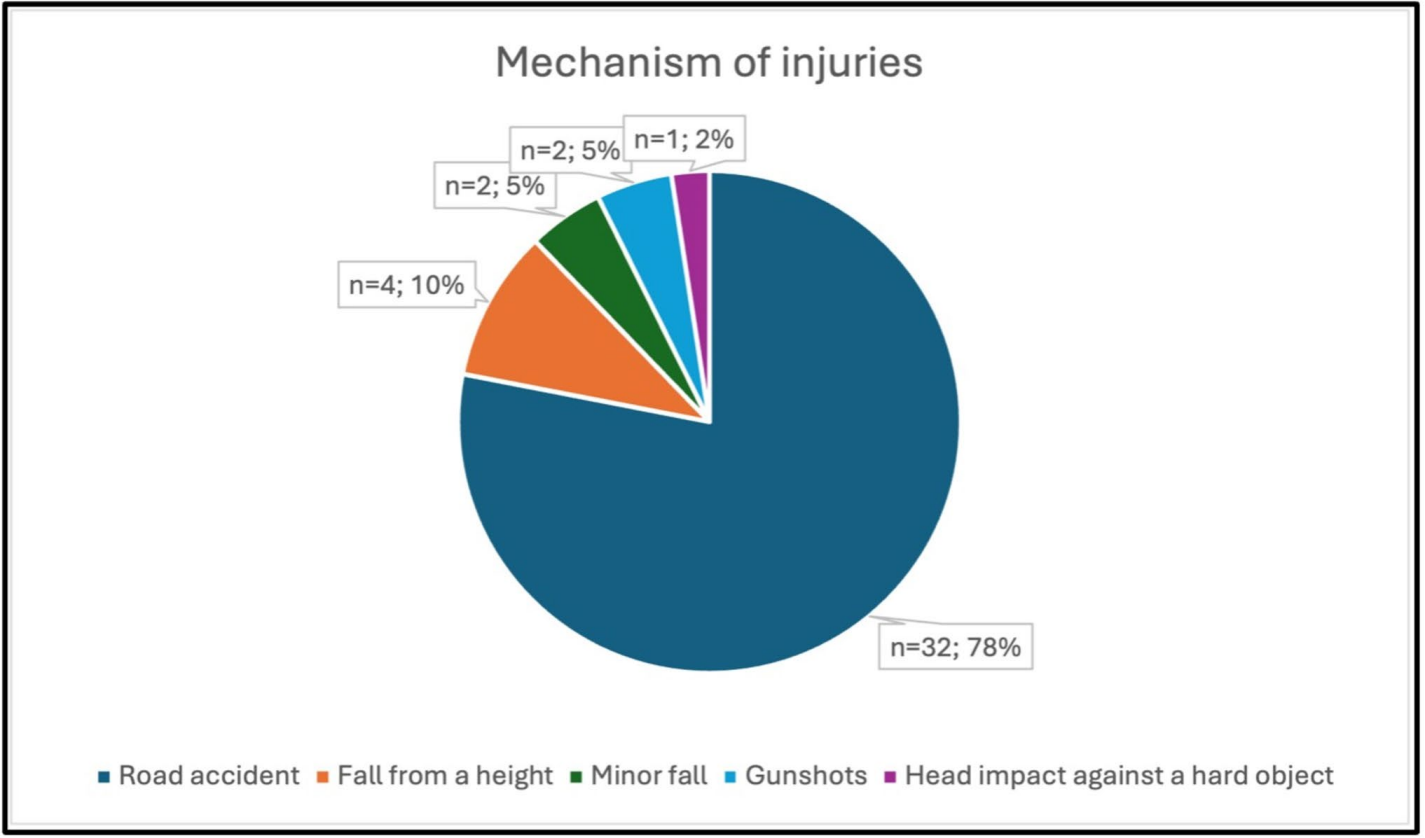
Mechanism of injuries in all patients

Regarding mechanisms of injury, 78.1% (*n* = 32) of patients had sustained traffic-related trauma (Fig. 2). Only one patient was asymptomatic at presentation. Altered consciousness was observed in 87.8% (*n* = 36) and convulsive seizures in 17.1% (*n* = 7). At least one abnormal physical examination finding was present in 87.8% of patients. The median Glasgow Coma Scale (GCS) score on admission was 5.0 (3.0–8.0), with 80.5% (n = 33) scoring below 9. Laboratory evaluation revealed ATC in 73% and DIC in 28.6% of patients (Table 1).

**Table 1:** Clinical characteristics of all patients

Clinical Characteristics	*n* (%)
Symptoms	
- Change of consciousness	36 (87.8)
- Convulsive seizure	7 (17.1)
- Vomiting	5 (12.2)
Physical Examination Findings	
- Abnormal pupillary light reflex	23 (56.2)
- Scalp incision	9 (22)
- Otorrhea	7 (17.1)
- Rhinorrhea	4 (9.8)
GCS Group	
- Mild (14—15)	6 (14.6)
- Moderate (9—13)	2 (4.9)
- Severe (< 9)	33 (80.5)
Lab Results^a^	
- ATC	27 (73)
- DIC	10 (28.6)
Intubation in ED	32 (78)
ICU admission	34 (82.9)
Mortality	14 (34.1)
Neurological status at discharge	
- Favourable (GOSE 6—8)	12 (31.6)
- Unfavourable (GOSE 1—5)	26 (68.4)

*GCS* Glasgow coma scale, *ATC* Acute traumatic coagulopathy, *DIC* Disseminated intravascular coagulation, *ED* Emergency department, *ICU* Intensive care unit, *GOSE* Glasgow outcome scale extended

^a^Due to deficiencies in laboratory parameters, ATC could be analysed in 37 patients and DIC in 35 patients

**Fig. 3:**
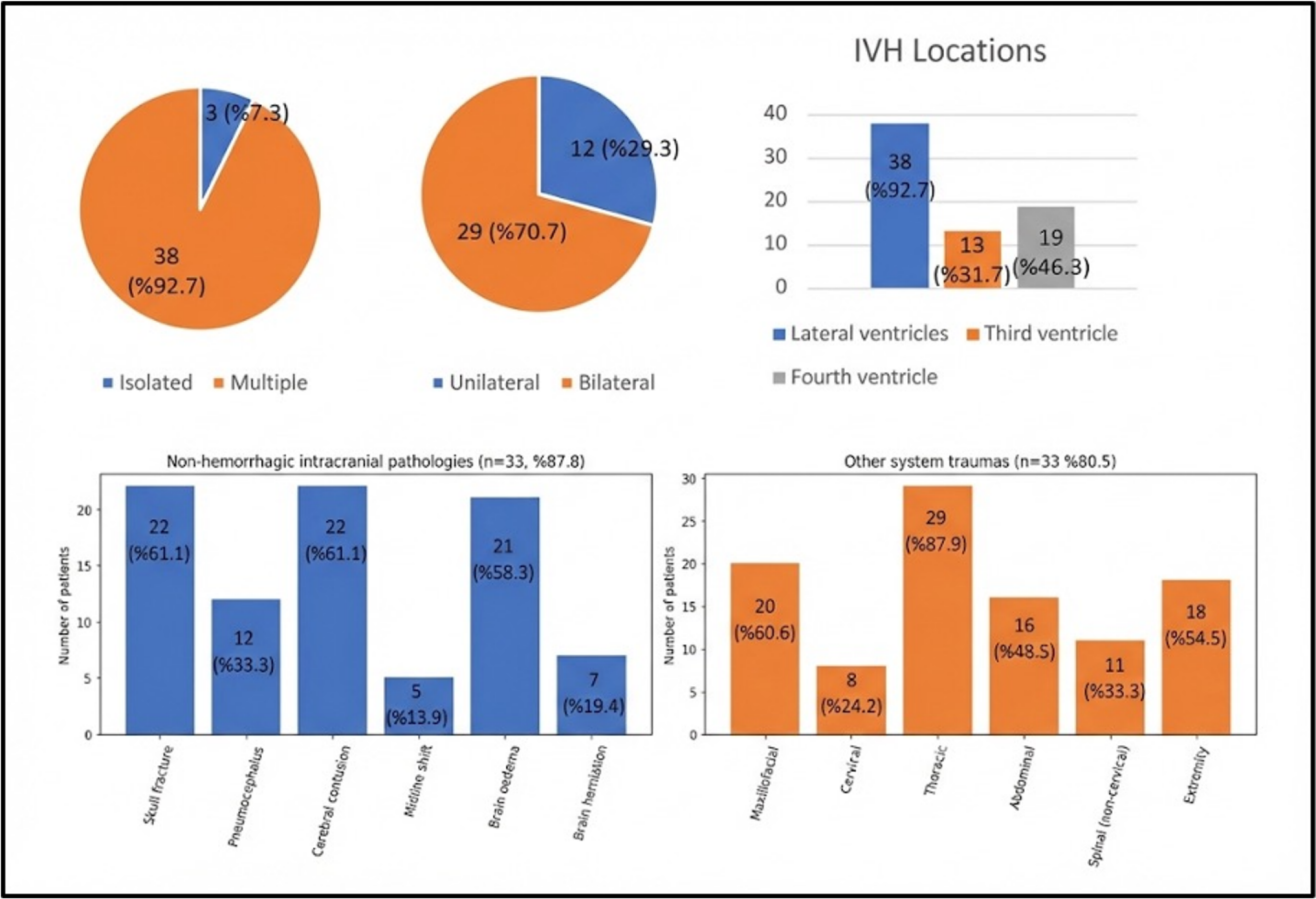
IVH characteristics, location, and additional injuries

Isolated tIVH was observed in only three patients (7.3%). Among patients with multiple intracranial haemorrhages (n = 38), subdural and intraparenchymal haemorrhages were the most common concomitant injuries (both n = 24, 63.2%), followed by subarachnoid haemorrhage (n = 23, 60.5%). Epidural haemorrhage was noted in only one patient. Bilateral haemorrhagic involvement was present in 70.7% of patients (n = 29), with the lateral ventricles most frequently affected (92.7%). Non-haemorrhagic intracranial pathology was observed in 87.8% (n = 36), and extracranial injuries in 80.5% (n = 33) (Fig. 3).

Regarding treatment, 33 patients (80.5%) received medical management alone, while six patients (14.6%) underwent a combination of medical and surgical treatment. External ventricular drainage for tIVH was performed in one patient, whereas the remaining five received surgery for concomitant intracranial injuries. Two patients were managed conservatively without intervention. Endotracheal intubation was required in 78% of patients (*n* = 32) in the emergency department, and 82.9% (*n* = 34) were subsequently admitted to the intensive care unit. The overall mortality rate was 34.1% (*n* = 14). GOSE scores at discharge were available for 38 patients (three were transferred to other centres), with only 31.6% (*n* = 12) achieving a good neurological outcome (Table 1).

In addition, hydrocephalus development was retrospectively assessed in 38 patients with available follow-up data. Hydrocephalus developed in 10 patients (26.3%) during follow-up. Of these, 3 patients (7.9%) required surgical intervention, including 1 patient who underwent external ventricular drainage in the acute phase and 2 patients who required ventriculoperitoneal shunt placement during follow-up.

Comparative analysis revealed that ATC, bilateral haemorrhages, concomitant subdural haemorrhage, cerebral oedema, and extracranial injuries were significantly associated with poor neurological outcome (*p* < 0.05). All patients with mild-to-moderate GCS at admission or isolated tIVH had favourable neurological outcomes. Conversely, all patients with DIC, midline shift, or brain herniation on neuroimaging had poor neurological outcomes (Table 2).

**Table 2:** Comparative analysis based on patients' neurological conditions

		Unfavourable *n* = 26 (%)	Favourable *n* = 12 (%)	*p* value
Age (years)^a^		13.8 (8.8—16.8)	12.6 (2.8—15.3)	0.354
GCS Group				
- Mild		-	6 (50)	N/A
- Moderate		-	1 (8.3)	
- Severe		26 (100)	5 (41.7)	
ATC^b^				
- No		4 (17.4)	6 (54.5)	0.045
- Yes		19 (82.6)	5 (45.5)	
DIC^b^				
- No		13 (61.9)	11 (100)	N/A
- Yes		8 (38.1)	-	
Unilateral haemorrhage		5 (19.2)	7 (58.3)	**0.026**
Bilateral haemorrhage		21 (80.8)	5 (41.7)	
Isolated IVH		-	3 (25)	N/A
Multiple haemorrhage		26 (100)	9 (75)	
In the multiple haemorrhage group (*n* = 35)	Subdural haemorrhage			
	- No	6 (23.1)	7 (77.8)	**0.006**
	- Yes	20 (76.9)	2 (22.2)	
	Subarachnoid haemorrhage			
	- No	11 (42.3)	4 (44.4)	1.0
	- Yes	15 (57.7)	5 (55.6)	
	Intraparenchymal haemorrhage			0.243
	- No	8 (30.8)	5 (55.6)	
	- Yes	18 (69.2)	4 (44.4)	
Non-haemorrhagic intracranial pathology				**0.027**
- No		1 (3.8)	4 (33.3)	
- Yes		25 (96.2)	8 (66.7)	
In the non-haemorrhagic intracranial pathology group (*n* = 33)	Skull fracture			
	- No	10 (40)	4 (50)	0.695
	- Yes	15 (60)	4 (50)	
	Pneumocephalus			
	- No	17 (68)	6 (75)	1.0
	- Yes	8 (32)	2 (25)	
	Cerebral contusion			
	- No	10 (40)	4 (50)	0.695
	- Yes	15 (60)	4 (50)	
	Midline shift			
	- No	20 (80)	8 (100)	N/A
	- Yes	5 (20)	-	
	Brain oedema			
	- No	8 (32)	6 (75)	**0.047**
	- Yes	17 (68)	2 (25)	
	Brain herniation			
	- No	18 (72)	8 (100)	N/A
	- Yes	7 (28)	-	
Concomitant injuries involving other organ systems				
- No		2 (7.7)	6 (50)	**0.007**
- Yes		24 (92.3)	6 (50)	

*GCS* Glasgow coma scale, *ATC* Acute traumatic coagulopathy, *DIC* Disseminated intravascular coagulation, *IVH* Intraventricular haemorrhage

^a^The data are presented as the median (25th–75th percentile)

^b^Among patients with available data about neurological outcome, due to deficiencies in laboratory parameters, ATC could be analysed in 34 patients and DIC in 32 patients

Mortality analysis showed that patients with DIC, fourth ventricular haemorrhage, concomitant subdural haemorrhage, skull fracture, brain herniation, maxillofacial, or abdominal trauma had significantly higher mortality rates (Table 3).

**Table 3:** Comparative analysis based on patients' survival rates

			Mortality *n* = 14 (%)	Survival *n* = 27 (%)	*p* value
Age (years)^a^			13.8 (9.3—16.9)	12.4 (3.8—15.5)	0.157
GCS Group					
- Mild			-	6 (22.2)	N/A
- Moderate			-	2 (7.4)	
- Severe			14 (100)	19 (70.4)	
ATC^b^					
- No			2 (16.7)	8 (32)	0.445
- Yes			10 (83.3)	17 (68)	
DIC^b^					
- No			5 (45.4)	20 (83.3)	**0.041**
- Yes			6 (54.5)	4 (16.7)	
Unilateral haemorrhage			3 (21.4)	9 (33.3)	0.494
Bilateral haemorrhage			11 (78.6)	18 (66.7)	
Isolated IVH			-	3 (11.1)	N/A
Multiple haemorrhage			14 (100)	24 (88.9)	
In the multiple haemorrhage group (*n* = 38)		Subdural haemorrhage			
		- No	2 (14.3)	12 (50)	**0.028**
		- Yes	12 (85.7)	12 (50)	
		Subarachnoid haemorrhage			
		- No	4 (28.6)	11 (45.8)	0.294
		- Yes	10 (71.4)	13 (54.2)	
		Intraparenchymal haemorrhage			
		- No	5 (35.7)	9 (37.5)	0.912
		- Yes	9 (64.3)	15 (62.5)	
Non-haemorrhagic intracranial pathology					
- No			1 (7.1)	4 (14.8)	0.645
- Yes			13 (92.9)	23 (85.2)	
In the non-haemorrhagic intracranial pathology group (*n* = 36)		Skull fracture			
		- No	2 (15.4)	12 (52.2)	**0.03**
		- Yes	11 (84.6)	11 (47.8)	
		Pneumocephalus			
		- No	7 (53.8)	17 (73.9)	0.281
		- Yes	6 (46.2)	6 (26.1)	
		Cerebral contusion			
		- No	7 (53.8)	7 (30.4)	0.166
		- Yes	6 (46.2)	16 (69.6)	
		Midline shift			
		- No	11 (84.6)	20 (87)	1.0
		- Yes	2 (15.4)	3 (13)	
		Brain oedema			
		- No	3 (23.1)	12 (52.2)	0.089
		- Yes	10 (76.9)	11 (47.8)	
		Brain herniation			
		- No	7 (53.8)	22 (95.7)	**0.005**
		- Yes	6 (46.2)	1 (4.3)	
Concomitant injuries involving other organ systems					
- No			1 (7.1)	7 (25.9)	0.227
- Yes			13 (92.9)	20 (74.1)	
Concomitant injuries involving other organ systems group (*n* = 33)	Maxillofacial trauma				
	- No		2 (15.4)	11 (55)	**0.023**
	- Yes		11 (84.6)	9 (45)	
	Cervical trauma				
	- No		10 (76.9)	15 (75)	1.0
	- Yes		3 (23.1)	5 (25)	
	Thoracic trauma				
	- No		-	4 (20)	N/A
	- Yes		13 (100)	16 (80)	
	Abdominal trauma				
	- No		2 (15.4)	15 (75)	**0.001**
	- Yes		11 (84.6)	5 (25)	
	Spinal trauma (non-cervical)				
	- No		8 (61.6)	14 (70)	0.714
	- Yes		5 (38.4)	6 (30)	
	Extremity trauma				
	- No		7 (53.8)	8 (40)	0.435
	- Yes		6 (46.2)	12 (60)	

*GCS* Glasgow coma scale, *ATC* Acute traumatic coagulopathy, *DIC* Disseminated intravascular coagulation, *IVH* Intraventricular haemorrhage

^a^The data are presented as the median (25th–75th percentile)

^b^Due to deficiencies in laboratory parameters, ATC could be analysed in 37 patients, and DIC in 35 patients

## Discussion

TBI is an increasingly prevalent cause of morbidity and mortality in paediatric patients [8]. tIVH is the least common type of intracranial haemorrhage in this population. In the study by Scurfield et al. tIVH was observed in only 3.6% of paediatric patients with abnormal CT findings or neurological deficits following head trauma [9]. Similarly, Shibahashi et al. reported tIVH in 1.6% of patients with intracranial injuries, and Atzema et al. reported it in 13.7% [10, 11]. As a regional referral paediatric emergency department managing the majority of trauma cases, we observed tIVH in 5.2% of patients with traumatic intracranial haemorrhage. Advances in diagnostic imaging, particularly CT, have increased detection rates of tIVH [12].

The pathophysiology of traumatic intraventricular haemorrhage (tIVH) is thought to involve several mechanisms, including sudden dilation of subependymal veins due to negative pressure during trauma (cavitation theory), shear injury between deep nuclei and perforating vessels, and damage caused by angular acceleration/deceleration forces. Studies have indicated that free movement of the head is a key factor in the development of shear injuries. Accordingly, tIVH is observed more frequently in traffic-related accidents, which induce rotational head movement, than in fall-related injuries [13–15]. In our study, the majority of patients with tIVH had sustained trauma in traffic accidents, supporting this hypothesis. Additionally, it has been shown that adjacent intraparenchymal haemorrhages can extend into the ventricular system and contribute to tIVH; however, this typically occurs 6–12 h post-trauma [16]. In our cohort, as brain CT was performed in the early post-traumatic period, we cannot comment on the contribution of this mechanism to the development of tIVH in our patients.

Isolated tIVH is rare and typically occurs in conjunction with other intracranial injuries. Atzema et al. reported that only 11.4% of paediatric patients with tIVH had isolated involvement. In a multicentre prospective study by Lichenstein et al. 18.9% of 53 patients with tIVH had isolated lesions, all of which presented with a GCS of 15, required neither surgery nor intensive care, and demonstrated no mortality [7, 11, 17]. These findings suggest that isolated tIVH represents a localized vascular injury with relatively mild intracranial impact. Accordingly, in paediatric patients with intracranial trauma, the presence of isolated tIVH can serve as a low-risk criterion for repeat imaging [18]. In our study, isolated tIVH was observed at a rate of 7.3%. Although this rate is lower than that reported in the literature, it may be attributable to the referral of more severe trauma cases to our center from the surrounding region. All isolated tIVH cases in our cohort had favourable outcomes, further supporting the notion that isolated tIVH is generally associated with a favourable clinical course.

Previous studies indicate that tIVH frequently coexists with cerebral contusions, subarachnoid haemorrhage, subdural haemorrhage, and intraparenchymal haemorrhage. The presence of concomitant intracranial pathologies may provide clues about the type and location of tIVH, as they are related to the velocity and direction of the traumatic force [10, 19, 20]. In our study, skull fractures and cerebral oedema were also frequently observed alongside tIVH. Similarly, Shibahashi et al. reported a higher incidence of extracranial injuries in patients with tIVH, most commonly thoracic trauma, consistent with our findings [10]. These observations collectively may suggest that tIVH serves as an indicator of trauma severity.

IVH is associated with high morbidity and mortality in both paediatric and adult populations. Scurfield, Atzema, and Lee reported that only 30–40% of patients achieved long-term favourable neurological outcomes [9, 11, 13]. Mortality rates range from 21 to 62% [9, 13, 20–22]. In our study, consistent with the existing literature, a favourable neurological outcome was observed in 31.6% of patients, while the mortality rate was 34.1%. However, the independent contribution of tIVH to poor outcomes remains unclear. Some studies suggest that rotational and shearing forces causing tIVH may also induce widespread neuronal injury and damage to the corpus callosum, contributing to a poor prognosis [23–25]. Conversely, isolated tIVH is associated with favourable outcomes, suggesting that adverse prognosis is primarily driven by the severity of trauma and concomitant injuries [11, 17, 20]. Shibahashi et al. similarly observed higher mortality among patients with tIVH, but multivariable analysis indicated that concomitant injuries, rather than tIVH itself, were the main determinant [10]. Our findings further support the growing body of evidence suggesting that adverse outcomes in tIVH are more closely related to the severity of concomitant injuries than to the presence of intraventricular haemorrhage itself.

Hydrocephalus is a well-recognised complication of intraventricular haemorrhage and an important determinant of clinical outcome [26, 27]. In our cohort, hydrocephalus was identified on follow-up imaging in 26.3% of patients with available data; however, only a small proportion (7.9%) required surgical intervention. Notably, no cases requiring treatment were observed among patients with isolated tIVH, suggesting that clinically significant hydrocephalus may be associated with more extensive or complex injury patterns. The discrepancy between the relatively high rate of radiologically detected hydrocephalus and the lower rate of intervention indicates that a substantial proportion of cases may be subclinical or not clinically consequential. Nevertheless, the occurrence of delayed ventriculoperitoneal shunt placement in two patients highlights the potential for late-onset hydrocephalus and underscores the importance of prolonged clinical and radiological follow-up. Overall, these findings suggest that while hydrocephalus is a relatively frequent radiological finding following tIVH, its clinical significance varies, and the need for intervention appears limited to a subset of patients. However, given the retrospective design and the absence of standardized follow-up imaging protocols, the true incidence, timing, and clinical relevance of hydrocephalus in this cohort may be subject to under- or overestimation.

Our study aimed to address gaps in the literature regarding prognostic factors in paediatric tIVH. Patients presenting with mild or moderate GCS and isolated tIVH had favourable outcomes. In contrast, coagulopathy, subdural haemorrhage, and brain herniation were significantly associated with poor neurological outcomes and mortality. Bilateral haemorrhage, cerebral oedema, and midline shift were linked to poor neurological outcome, while fourth ventricular haemorrhage, skull fracture, maxillofacial trauma, and abdominal trauma were associated with increased mortality. Extracranial injuries, particularly abdominal trauma, may serve as markers of high-energy trauma contributing to increased mortality. These findings, though not previously analysed specifically for paediatric tIVH, align with prognostic trends in severe TBI. Supporting this, in a study by LeRoux et al. involving 43 mostly adult patients with tIVH, advanced age, low admission GCS, and haemorrhage involving all ventricles were found to be associated with poor neurological outcomes [19].

TBI is generally classified as severe, moderate, or mild based on the patient’s GCS score. Multiple studies have demonstrated that admission GCS, particularly the motor component, serves as an independent prognostic factor in pediatric TBI [28–31]. Trauma-induced acute coagulopathy is increasingly recognised as a critical predictor of poor outcome in pediatric TBI [32]. Studies have shown that it is associated with both increased mortality and poor neurological outcomes, whether in isolated head trauma or multiple injuries [33–35]. In our cohort, patients meeting ISTH criteria for DIC exhibited significantly higher risks of both mortality and poor neurological outcomes.

Acute subdural haemorrhage in patients with TBI is associated with unexpected mortality and poor neurological outcomes, independent of age or GCS scores, and is considered one of the most critical subtypes linked to poor prognosis [36, 37]. Kaewborisutsakul et al. reported that the presence of concomitant tIVH in children with traumatic subdural haemorrhage increased the risk of poor neurological outcomes sixfold [38]. In our study, we further examined the interaction of these two haemorrhage types from another perspective and found that, in children with tIVH, the presence of an accompanying subdural haemorrhage significantly increased both mortality and the risk of poor neurological outcomes.

Brain herniation results from increased intracranial pressure due to a large supratentorial space-occupying lesion or cerebral oedema and can rapidly lead to death through brainstem ischemia. It was previously considered an invariably fatal clinical finding; however, case series have reported successful treatment of brain herniation following early neurosurgical interventions, particularly decompressive craniectomy [39]. Studies indicate that mortality in TBI patients with herniation ranges from 30–60%, while favourable neurological outcomes are observed in 18–34% of cases [39–41]. In our study, no cases of brain herniation occurred in patients with isolated tIVH. Among the seven patients with both tIVH and herniation, six succumbed to their injuries and the sole survivor was discharged with a poor neurological outcome (GOSE 3).

Imaging-based scoring systems, such as the Rotterdam and Marshall CT scores, assess key radiological features including basal cistern compression, midline shift, the presence of epidural mass lesions, and intraventricular or subarachnoid haemorrhage. The prognostic value of these scoring systems has been well established in pediatric TBI (42, 43). In line with these scoring principles, our findings demonstrate that bilateral haemorrhagic involvement, cerebral oedema, and midline shift are significantly associated with poor neurological outcomes in pediatric patients with tIVH. These radiological features likely reflect the severity and diffuseness of brain injury rather than the isolated effect of tIVH itself, suggesting that tIVH may represent a marker of high-energy trauma rather than an independent pathological entity.

This study has several limitations. It is a single-centre, retrospective analysis, and tIVH is inherently rare in paediatric populations. The predominance of non-isolated tIVH cases limited our ability to evaluate the independent prognostic impact of tIVH and may have introduced confounding by injury severity. Hydrocephalus assessment was retrospective and based on available clinical and imaging data, and the absence of standardised follow-up may have led to underestimation of its incidence. Although it was insufficient to conduct a robust multivariable logistic regression analysis for independent risk factors, our sample size with 84.88% post-hoc power is relatively large compared to previous reports.

## Conclusion

Traumatic intraventricular haemorrhage in children is a rare clinical condition that most commonly occurs in the setting of significant head trauma. In this study, we identified several clinical and radiological factors associated with mortality and poor neurological outcomes in paediatric patients with tIVH, including low admission GCS, coagulopathy, and the presence of concomitant intracranial and extracranial injuries. In contrast, patients with isolated tIVH generally demonstrated favourable outcomes. These findings underscore the importance of comprehensive clinical and radiological assessment for early risk stratification and management in children with tIVH, particularly in the presence of associated injuries.

## Data Availability

No datasets were generated or analysed during the current study.

## Abbreviations

ATC: Acute traumatic coagulopathy
CT: Computed tomography
DIC: Disseminated intravascular coagulation
GCS: Glasgow Coma Scale
GOSE: Glasgow Outcome Scale-Extended
ICU: Intensive Care Unit
INR: International normalized ratio
ISTH: International Society on Thrombosis and Haemostasis
SPSS: Statistical Package for the Social Sciences
TBI: Traumatic brain injury
tIVH: Traumatic intraventicular haemorrhage
